# Prolonged viral positivity induced recurrent coronavirus disease 2019 (COVID-19) pneumonia in patients receiving anti-CD20 monoclonal antibody treatment: Case reports

**DOI:** 10.1097/MD.0000000000028470

**Published:** 2021-12-30

**Authors:** Burak Deveci, Rabin Saba

**Affiliations:** a Medstar Antalya Hospital, Hematology and Stem Cell Transplantation Unit, Antalya, Turkey; b Medstar Antalya Hospital, Infectious Disease Unit, Antalya, Turkey.

**Keywords:** coronavirus disease 2019 pneumonia, humoral immunity, obinutuzumab, rituximab

## Abstract

**Introduction:**

The outbreak of novel coronavirus (severe acute respiratory syndrome coronavirus 2), which causes the coronavirus disease 2019 (COVID-19), is the most important current health problem. The number of patients is increasing worldwide. Pneumonia is the most life-threatening complication of the disease. Prolonged viral shedding in hematological patients with COVID-19 has been demonstrated; however, data on COVID-19 patients receiving anti-CD20 monoclonal antibody therapy are limited. Accordingly, focusing on humoral immunity, herein, we present 4 COVID-19 patients who were on anti-CD20 monoclonal antibody treatment and had prolonged pneumonia.

**Patient concerns:**

Two of 4 patients were on rituximab and the other 2 were on obinutuzumab therapy.

**Diagnosis:**

The polymerase chain reaction test results for severe acute respiratory syndrome coronavirus 2 were positive for all 4 patients and their COVID pneumonia lasted for >50 days.

**Interventions:**

Although all patients were treated with an adequate amount of convalescent plasma, prolonged polymerase chain reaction positivity and prolonged pneumonia were possibly due to the lack of ability of the immune system to initiate its antibody response.

**Outcomes:**

Despite the administration of standard therapies, recurrent pneumonia observed in the present case series of non-neutropenic patients, in whom primary malignancies were under control.

**Conclusions:**

It is suggested that further investigations should be performed to understand the underlying pathophysiology.

## 1 Introduction

The outbreak of novel coronavirus (severe acute respiratory syndrome coronavirus 2 [SARS-CoV-2]), which causes the coronavirus disease 2019 (COVID-19), is the most important current health problem. According to data reported by the World Health Organization (WHO), >105,000,000 people were infected with COVID-19 and >2,250,000 of them died due to the disease and its complications as of February 2021.^[1]^

Patients with COVID-19 present within a wide range of distinct clinical entities from asymptomatic disease to mild to severe involvement with several evolutionary stages. Pneumonia is one of the most life-threatening complications in COVID-19.^[2]^ According to the WHO data, COVID-19 related death rate is approximately 2% worldwide; however, in a study conducted in China, the death rate was reported as at least 4% in patients with COVID-19 pneumonia.^[3]^ Patients with hematological malignancies are among the vulnerable groups to COVID-19 due to immunosuppression resulted from underlying diseases and drugs used to treat malignancy.[^2^,^4^,^5^]

In a case report by Karataş et al,^[6]^ prolonged viral shedding was reported in a patient with hematological malignancy undergoing autologous stem cell transplantation; however, there is no enough data on COVID-19 patients receiving anti-CD20 monoclonal antibody treatment.^[6]^ Accordingly, herein, we present 4 COVID-19 pneumonia cases with B lymphocyte malignancies who were receiving anti-CD20 monoclonal antibody treatment.

## 2 Case series

### 2.1 Case 1

A 34-year-old non-smoker female patient with the diagnosis of stage IV-B grade 3a follicular lymphoma without any other comorbidities was admitted to the emergency service with the complaints of fever, shortness of breath, and cough. She was febrile with a body temperature of 38.7 °C and her oxygen saturation was 85% on room air. Her polymerase chain reaction (PCR) test result for SARS-CoV-2 was positive. Her computer tomography (CT) scan revealed bilateral pneumonic infiltration suggesting COVID-19 pneumonia. Her laboratory results showed hypogammaglobinemia (an immunoglobulin [Ig] G [IgG] level of 319 mg/dL) but no neutropenia. On her medical history, she was first diagnosed with lymphoma 14 months ago and was still on remission with rituximab maintenance treatment. Based on the treatment protocol, rituximab was administered with a dose of 375 mg/m^2^ every 3 months, the last dose being administered 34 days before her admission to the hospital.

During hospitalization, the patient was initiated on standard antiviral therapy (favipiravir), convalescent plasma therapy, intravenous immunoglobulin (IVIG; 400 mg/kg for 1 day) and parenteral antibiotics for bacterial co-infections; supportive therapies (such as oxygen support, positioning, and antipyretics) were also given. She was afebrile by the end of the 2nd day of hospitalization. Respiratory distress requiring at least 4 L/min oxygen inhalation continued for 10 days. Although the second PCR test result of the patient was still positive on day 12, she was discharged without any complaints on day 14.

Eleven days after discharge (25 days after the first PCR positivity), the patient presented with the same initial symptoms started one day ago. Her CT scan revealed a marked progression in pneumonic infiltration. The patient was re-hospitalized and the same treatment protocol was initiated. Observing rapid progression of pulmonary infiltrations under antiviral and antibiotic therapy, recurrent disease was suspected. A percutaneous lung biopsy was performed. Pathologic findings revealed pneumonia without malignant infiltration. In addition, the PCR test result was still positive on day 38 after the first PCR positivity.

During the hospitalization, the patient did not suffer from any complications. Acute phase reactants became normal in 10 days and her complaints were completely resolved. She was hospitalized for 22 days and her PCR test result was still positive on discharge.

Seven days after her second discharge, she was admitted to the hospital for the third time (54 days after her first PCR positivity) with the same initial complaints. She had an unexpected prolonged PCR positivity and her anti-SARS CoV-2 IgG and immunoglobulin M (IgM) antibodies were negative. On the other hand, her thorax CT scan showed significant improvement in pneumonic infiltration. She was discharged on the 68th day of initial diagnosis with normal acute phase reactant levels and PCR positivity but without any complaints. During follow up patient had near complete remission and maintenance therapy was withdrawn after 7th course to avoid infectious complications.

### 2.2 Case 2

A 45-year-old nonsmoker female patient with the diagnosis of Binet stage C chronic lymphocytic leukemia without any other comorbidities was admitted to the hematology outpatient clinic with the complaints of sore throat, fever, and cough. Her PCR test result for SARS-CoV-2 was positive. The patient was first diagnosed with chronic lymphocytic leukemia in August 2013 and she was still on rituximab plus bendamustine treatment on admission and her last rituximab treatment was administered 29 days before the PCR positivity. She did not experience any shortness of breath and her oxygen saturation was 97% on room air. Her CT scan revealed mediastinal enlarged lymph nodes without any pneumonic infiltration. She had hypogammaglobinemia (an IgG level of 387 mg/dL) but no neutropenia. Since she had no indication for hospitalization, favipiravir, low molecular weight heparin, and paracetamol was prescribed and the patient was sent home after IVIG replacement. Her chemo-immunotherapy protocol was postponed.

The patient was re-admitted to emergency service after 24 days of her initial admission with the same initial symptoms. She was dyspneic with oxygen saturation of 82% on room air and febrile with a body temperature of 38.8 °C. A low-dose thorax CT scan revealed bilateral pneumonic infiltration that was strongly suggestive of COVID-19 pneumonia. The patient was hospitalized and initiated on supportive therapies (such as oxygen, positioning, antipyretics), standard antiviral therapy (favipiravir), convalescent plasma therapy, and parenteral antibiotics for bacterial co-infections. The patient was hospitalized for 22 days and discharged without any complaints. Her PCR test was still positive on the day of discharge (46 days after the first positive PCR test).

On the control visit 1 week after discharge, the patient had complaints of cough and dyspnea. The control CT scan showed marked progression of pneumonic infiltration and the PCR test result was still positive (53 days after the first positive PCR test). The patient was hospitalized for 20 days due to COVID-19 pneumonia. The patient was discharged after clinical recovery; however, she still had a positive PCR test result (73 days after the first positive PCR test) and her anti-SARS-CoV-2 antibodies were negative on the day of discharge. During follow up patient's IGHV was detected as unmutated and her therapy was planned to continue with ibrutinib.

### 2.3 Case 3

A 62-year-old ex-smoker male patient with stage IV mantle cell lymphoma who was receiving obinutuzumab and bendamustine was admitted to our hospital with fever and dyspnea. The patient reported that his last chemo-immunotherapy was administered 19 days ago. The patient had hypertension and type 2 diabetes mellitus, both of which were well controlled with oral drugs. Initial physical examination findings revealed that the patient had a body temperature of 39.2 °C, respiratory distress, and hypotension. The patient was neutropenic (an absolute neutrophil count of 90 cells/mm^3^) and his PCR test result for SARS-CoV-2 virus was positive. The CT scan of thorax revealed findings compatible with COVID-19 pneumonia, accompanied by probable bacterial pneumonia.

The patient was hospitalized and initiated on standard therapies for COVID-19, bacterial pneumonia, and neutropenic fever. The patient had no life-threatening complications during hospitalization and was discharged on day 18 based on clinical recovery and marked radiological improvement; however, his PCR test was still positive.

The patient was readmitted with fever 11 days after discharge and his CT scan showed progression in pneumonic infiltration. During his second hospitalization (22 days), the findings of all bacterial cultures were negative and the patient never became neutropenic. The PCR test result was still positive for the patient and his anti-SARS-CoV-2 antibodies were negative on the day of discharge (day 51). During follow up patient was not in remission and his therapy was planned to continue with ibrutinib.

### 2.4 Case 4

A 66-year-old male patient with the diagnosis of relapsed and refractory follicular lymphoma, who was receiving obinutuzumab and bendamustine as the fifth-line therapy without autologous stem cell transplant that could not be performed because of mobilization failure, was admitted with the complaints of cough and fever. His medical history otherwise was unremarkable except for hypertension and the last treatment protocol with obinutuzumab was administered 22 days ago. His PCR test result for SARS-CoV-2 was positive and his thorax CT scan revealed findings compatible with COVID-19 pneumonia. The patient was not neutropenic but had hypogammaglobinemia (IgG: 317 mg/dL). The patient was hospitalized for 12 days in the intensive care unit, followed-up for 11 days in the COVID-19 clinic, and then discharged based on clinical recovery; however, his PCR test finding was still positive.

Two weeks later, the patient was admitted with the same initial symptoms. He had no signs of bacterial infection or lymphoma progression; however, the CT findings showed viral pneumonia. The blood cultures and tests for other respiratory tract viruses did not reveal any bacteria or viral antigens except for SARS-Cov-2. Although the patient required noninvasive respiratory support, he was never intubated. During 2 hospitalization periods, antibody tests performed for 4 times both for IgM and for IgG yielded negative results. Convalescent plasma and IVIG were added to standard therapies. The patient was hospitalized for 26 days and then discharged on the day 61 after the first PCR test positivity and was negative for anti-SARS-CoV-2 antibodies. During follow up patient's therapy was planned to continue with ibrutinib.

## 3 Discussion

In these case series, focusing on the importance of humoral immunity in COVID-19, we reported 4 patients who were on anti-CD20 monoclonal antibody treatment for their hematological disorder and had recurrent COVID-19 pneumonia and prolonged PCR positivity for SARS-CoV-2. A summary of general characteristics of the patients are presented in Table 1.

**Table 1:** Summary of the general characteristics of the cases.

	Case 1	Case 2	Case 3	Case 4
Age, y	34	45	62	66
Sex	Female	Female	Male	Male
Type of blood malignancy	FL (Stage IV-B grade 3a)	CLL (Binet stage C)	MCL (Stage IV)	FL
Comorbidities	No	No	HT, T2D (controlled)	HT (controlled)
Type of anti-CD20	Rituximab	Rituximab+bendamustine	Obinutuzumab	Obinutuzumab
The time interval from the last dose of anti-CD20 monoclonal antibody to the COVID-19 positivity, d	34	24	19	22
Neutropenia	No	No	Neutropenic during the 1st admission (an absolute neutrophil count of 90 cells/mm^3^) Not neutropenic during the 2nd admission	No
Lymphocyte count (10^3^/μL)	0.74	0.86	0.67	1.79
HGG	Yes IgG level of 319 mg/dL	Yes IgG level of 387 mg/dL	–	Yes IgG level of 319 mg/dL
PCR test positivity for SARS CoV-2, days	68	73	51	61
Antibodies for SARS CoV-2 on the day of discharge	Negative	Negative	Negative	Negative

Pneumonia is one of the most life-threatening complications in COVID-19.^[2]^ Since there is no current standard therapy for COVID-19, supportive therapeutic approaches, immune modulators, and some antiviral agents such as favipiravir and remdesivir are recommended. Although these therapeutic options can provide some clinical improvement, the mortality rate is still high in patients with pneumonia worldwide. In this case series, despite the administration of standard therapies, recurrent pneumonia was observed in our non-neutropenic patients whose primary malignancies were under control.

Anti-CD20 monoclonal antibody therapy is the most promising therapeutic approach in B cell malignancies such as chronic lymphocytic leukemia and B cell lymphomas. The most widely used drugs of this group are rituximab and obinutuzumab. Both drugs are potent B lymphocyte inhibitors that act through blocking CD20 molecules on B lymphocytes and which in turn strongly suppress the antibody response of the immune system.[^7^,^8^] In this case series, we aimed to focus on the importance of humoral immunity in COVID-19. Our patients with lymphoid malignancies had several common clinical situations. Among these, the most intriguing one was patients’ being on anti-CD20 monoclonal antibody therapy that caused hypogammaglobinemia and suppression of antibody response. However, all of patients were treated with convalescent plasma which consisted of adequate amounts of anti-SARS COV-2 IgG antibodies. Therefore, it can be assumed that early antibody response (IgM) may have a key role in viral clearance.

In their study, Xu et al^[9]^ reported several factors including male sex, advanced age, and systemic comorbidities (hypertension) that are related to prolonged viral shedding. In this case series, however, 2 patients were younger than 50 years of age, 2 of them were women, and none of them had uncontrolled comorbidity (Table 1). Therefore, although COVID-19 is the most contagious in 10 to 14 days of infection according to available data, isolation should be prolonged for those patients receiving anti-CD20 therapy.^[9]^

In our case series, we experienced some limitations in the management of our patients. One of these limitations was that the amount of anti-SARS CoV-2 antibodies in convalescent plasma could not be measured. Convalescent plasma was collected from seropositive (qualitative) donors 4 weeks after the PCR negativity. Therefore, the exact amount of specific antibodies (IgG and IgM) in convalescent plasma was not known. However, according to our current knowledge, IgM antibodies are supposed to either remain in a very low concentration or disappear in 4 weeks. The second limitation was switching antiviral drugs. Remdesivir is one of the promising antiviral agents in the treatment of COVID-19.^[10]^ However, it was not available while these 4 patients were being treated. If remdesivir could have been provided for these patients, they might have had the chance of shifting to the PCR negativity. The third limitation was, we could not compare these 4 patients with patients with B-cell lymphoproliferative disorders receiving anti-CD20 monoclonal antibody therapy and had COVID-19, but fully recovered from SARS-CoV-2 infection uneventfully because those patients in the second group were followed at home under isolation.

Although our patients were not neutropenic, 3 of them had lymphopenia which is probably due to anti-CD20 therapy and can be another factor for hypogammaglobinemia and prolonged viral positivity. On the other hand, we know that anti-CD20 therapies may cause lymphopenia and especially decrease in CD4+ T cells.[^11^,^12^] So this can be another cause underlying prolonged viral positivity.

Ibrutinib seems to be one of the safest agents during this COVID outbreak. According to our current knowledge; use of Bruton Tyrosine kinase (BTK) inhibitors can impair some functions of the innate immunity and increases the susceptibility to infections or impaired humoral immunity in patients.^[13]^ Two pilot studies those were conducted on B cell malignancies revealed that BTK inhibition may have protective effects against SARS-CoV2 virulence.[^14^,^15^]

In conclusion, despite the administration of standard therapies, recurrent pneumonia observed in the present case series of non-neutropenic patients, in whom primary malignancies were under control, suggested that further investigations should be performed to understand the underlying pathophysiology. Although all patients were treated with an adequate amount of convalescent plasma, PCR positivity for SARS CoV-2 and pneumonia prolonged possibly due to the lack of ability of the immune system to initiate its antibody response in patients under anti-CD20 therapies. Accordingly, clinicians should be aware of recurrent COVID-19 pneumonia and prolonged viral shedding in patients under anti-CD20 therapies.

## Acknowledgments

All authors want to thank Prof Dr Ihsan Karadoğan (deceased) for his scientific support.

## Author contributions

**Conceptualization:** Burak Deveci, Rabin Saba.

**Data curation:** Burak Deveci, Rabin Saba.

**Formal analysis:** Burak Deveci, Rabin Saba.

**Funding acquisition:** Burak Deveci, Rabin Saba.

**Investigation:** Burak Deveci, Rabin Saba.

**Methodology:** Burak Deveci, Rabin Saba.

**Writing – original draft:** Burak Deveci, Rabin Saba.

**Writing – review & editing:** Burak Deveci, Rabin Saba.
